# Association of Fluoride Biomarker Levels With Osteoarthritis Severity: A Cross-Sectional Study From a Fluorosis-Endemic Region in India

**DOI:** 10.7759/cureus.91606

**Published:** 2025-09-04

**Authors:** Sandip Dhole, Bhushan Kamble, Rohit Saluja, Aparna Varma, Abhishek J Arora, Sakthi Das

**Affiliations:** 1 Physical Medicine and Rehabilitation, All India Institute of Medical Sciences, Hyderabad, IND; 2 Community Medicine and Family Medicine, All India Institute of Medical Sciences, Hyderabad, IND; 3 Biochemistry, All India Institute of Medical Sciences, Hyderabad, IND; 4 Radiodiagnosis, All India Institute of Medical Sciences, Hyderabad, IND

**Keywords:** environmental health, fluoride biomarkers, kellgren-lawrence, osteoarthritis, reverse osmosis water, serum fluoride

## Abstract

Background: Osteoarthritis (OA) is a major cause of disability globally. Recent evidence suggests a potential role of environmental fluoride in musculoskeletal degeneration.

Objective: To evaluate the association between fluoride levels in serum, urine, and drinking water and the severity of OA in adults from a fluorosis-endemic region.

Methods: In this cross-sectional study, 119 adults with radiologically confirmed knee OA from Yadadri-Bhuvanagiri, Telangana, were evaluated. OA severity was graded using the Kellgren-Lawrence (KL) system. Pain and function were assessed using the visual analog scale (VAS) and the knee injury and osteoarthritis outcome score (KOOS). Fluoride levels in serum, urine, and household drinking water were measured as biomarkers of systemic and environmental fluoride exposure.

Results: Moderate-to-severe OA (KL grades 3-4) was found in 60 (50.4%) participants. Significantly higher serum fluoride levels were observed in this group (p=0.007). The use of untreated underground water was associated with advanced OA (OR: 3.6; CI: 1.3-9.8). Water purification using reverse osmosis systems was associated with better VAS (p=0.02) and KOOS scores (p=0.05).

Conclusion: Higher fluoride biomarker levels, especially in serum, are significantly associated with greater OA severity. Public health strategies focusing on safe water access and routine fluoride surveillance may mitigate OA burden in endemic areas.

## Introduction

Osteoarthritis (OA) is the most common chronic joint disorder globally and a major contributor to disability among older adults. It is characterized by progressive degeneration of articular cartilage, subchondral bone remodeling, osteophyte formation, and synovial inflammation, resulting in pain, stiffness, reduced mobility, and compromised quality of life [1,2]. According to the Global Burden of Disease (GBD) Study 2019, 528 million people worldwide were living with OA, and knee OA alone accounted for an age-standardized YLD rate of 138.2 per 100,000 population. Moreover, 22.4% of YLDs due to knee OA were attributable to high BMI, highlighting the significant role of modifiable metabolic risk factors [3].

While traditional risk factors such as mechanical loading, age, obesity, trauma, and genetic predisposition are well documented [4], increasing attention is being paid to environmental and metabolic influences, particularly fluoride exposure, in the development and progression of OA. Choubisa documented widespread dental and skeletal fluorosis in southern Rajasthan, highlighting the severity of endemic fluoride exposure in rural Indian populations [5].

Fluoride, a naturally occurring element found in rocks and groundwater, helps prevent dental caries at optimal concentrations (0.7-1.0 mg/L) as per WHO guidelines [6]. However, in many rural Indian communities, chronic exposure to elevated fluoride levels can lead to dental and skeletal fluorosis, as documented in clinical and neurological studies [7]. Skeletal fluorosis is a progressive condition characterized by excessive fluoride deposition in bones and cartilage, leading to increased bone density, calcified ligaments, restricted mobility, and, in severe cases, neurological impairment [8].

Beyond overt skeletal fluorosis, subclinical fluoride toxicity has been implicated in accelerating degenerative joint changes that may mimic or coexist with OA. Fluoride has been shown to disrupt chondrocyte metabolism, promote apoptosis, induce oxidative stress, and interfere with extracellular matrix synthesis, mechanisms implicated in OA progression [9,10]. OA pathology has been extensively modeled in animal and tissue-engineered systems, highlighting features such as cartilage thinning and matrix loss [11].

Epidemiological studies from China, Mexico, and India have observed increased prevalence of joint pain and OA-like symptoms in populations exposed to high fluoride concentrations, even in the absence of classical skeletal fluorosis [12]. However, few clinical studies have systematically evaluated the relationship between systemic fluoride biomarkers, such as serum and urine fluoride levels, and the severity of OA, particularly among Indian populations residing in fluoride-endemic regions. In Telangana, India, several rural communities depend on underground water sources that often contain fluoride concentrations exceeding the World Health Organization's (WHO) permissible limit of 1.5 mg/L, placing residents at risk for both skeletal fluorosis and potentially accelerated OA progression. Yadadri-Bhuvanagiri and nearby districts are such high-risk areas. In this context, the present study was conducted with three primary objectives: (1) to quantify fluoride exposure through measurement of fluoride levels in serum, urine, and drinking water; (2) to assess the association between these fluoride biomarkers and radiographic severity of knee OA using the Kellgren-Lawrence (KL) grading system; and (3) to evaluate the clinical burden of OA in terms of pain and functional impairment using the visual analog scale (VAS) and the knee injury and osteoarthritis outcome score (KOOS) [13,14,15]. This investigation aims to fill a significant gap in the intersection of environmental exposure and musculoskeletal health, highlighting the potential need for public health interventions that incorporate fluoride exposure monitoring in OA risk assessment and management strategies.

## Materials and methods

Study design and setting

This was a cross-sectional observational study conducted in the Yadadri-Bhuvanagiri and nearby districts in Telangana, India, a region known for its fluoride-endemic groundwater. The study was carried out over a 12-month period from January 2024 to December 2024 in the outpatient departments of local primary and secondary care hospitals.

Study population

Assuming a conservative prevalence p=50% (maximum variance), two-sided α=0.05 (Z=1.96), and desired absolute precision d=5%, the required sample size was 385. A total of 119 adult patients with knee pain and radiological evidence of OA were consecutively recruited during the study period due to feasibility constraints. With n=119 and the same conservative p=50%, the achieved precision was 9%, which we considered acceptable for this observational study. Radiographic confirmation of OA was based on the KL grading system.

Participants aged between 40 and 60 years with clinical symptoms of knee OA for eight weeks, such as pain, stiffness, or reduced joint function, and radiological evidence of OA (KL grade 1-4) were included in the study. Patients were excluded if they had diagnosed inflammatory arthritis (e.g., rheumatoid arthritis), a history of significant knee trauma or surgery, known metabolic bone diseases such as Paget’s disease or osteomalacia, or chronic renal failure or other conditions known to affect fluoride metabolism.

Data collection

Data collection was performed using a structured case record form. Demographic details, including age, sex, occupation, and education level, were noted. Lifestyle variables such as dietary habits, source of drinking water, use of reverse osmosis (RO) filters, smoking, alcohol use, and physical activity were documented. Clinical parameters included history of comorbidities (e.g., diabetes, hypertension), family history of OA, and BMI.

The KL grading system was used to classify the severity of radiographic knee OA (grades 0 to 4) [13]. The VAS was used to quantify knee pain intensity, with scores ranging from 0 (no pain) to 10 (worst pain) [14]. Functional outcomes were assessed using the KOOS, which evaluates pain, symptoms, activities of daily living, sport and recreation function, and knee-related quality of life [15].

Laboratory investigations

Venous blood and spot urine samples were collected from all participants under aseptic conditions. Drinking water samples were obtained from the participants’ primary household source. Fluoride concentration in serum, urine, and water was measured using a fluoride ion-selective electrode (ISE) method following standard protocols [16]. Fluoride levels were expressed in milligrams per liter (mg/L).

Statistical analysis

Data analysis was performed using SPSS version 26.0 (IBM Corp., Armonk, NY, USA). Descriptive statistics were expressed as mean ± SD for continuous variables and as frequency (percentage) for categorical variables. The chi-square test was used to assess associations between categorical variables. Normality was checked using the Shapiro-Wilk test. The Mann-Whitney U test was applied for non-normally distributed continuous variables. Logistic regression analysis was used to estimate odds ratios (OR) with 95% CI for the association between fluoride exposure and moderate-to-severe OA (KL grades 3-4). A p-value <0.05 was considered statistically significant.

Ethical considerations

The study was approved by the Institutional Ethics Committee of All India Institute of Medical Sciences (AIIMS), Bibinagar (approval number: AIIMS/BBN/IEC/JAN/396). Informed consent was obtained from all participants before enrollment.

## Results

Demographics and clinical characteristics

The study included 119 participants, of whom 105 (88.2%) were female. The majority were semi-skilled (68, 56.7%) or unskilled workers (35, 29.7%). The mean age was 50.6 years (SD: 5.8). A mixed diet was predominant among the participants (116, 97.5%). Table 1 summarizes the demographic and clinical characteristics.

**Table 1:** Demographics and clinical characteristics of the sample population (n=119) *KL grading system adapted from Kellgren JH, Lawrence JS. Ann Rheum Dis. 1957;16(4):494-502. ^#^VAS methodology based on Huskisson EC. Lancet. 1974;2(7889):1127-1131. VAS, visual analog scale; RO, reverse osmosis; OA, osteoarthritis; KL, Kellgren-Lawrence

Variables	Classification	N	%
Age (years), mean (SD)=50.6 (5.5)	40-45	25	21.0
	46-50	36	30.3
	51-55	30	25.2
	56-60	28	23.5
Gender	Male	14	11.8
	Female	105	88.2
Occupation	Unskilled	35	29.7
	Semi-skilled	68	56.7
	Skilled	10	8.5
	Highly-skilled	5	4.2
	Professional	1	0.9
Drinking water source	River with RO	20	17.0
	Lake with RO	1	0.9
	Underground with RO	92	77.0
	Underground without RO	6	5.1
Diet	Veg	3	2.5
	Mixed	116	97.5
Family H/o OA	Yes	30	25.2
	No	89	74.8
KL grade of OA*	1	9	7.6
	2	50	42.0
	3	43	36.1
	4	17	14.3
VAS score^#^	1-3	63	52.9
	4-5	34	28.6
	6-7	22	18.5

**Figure 1:**
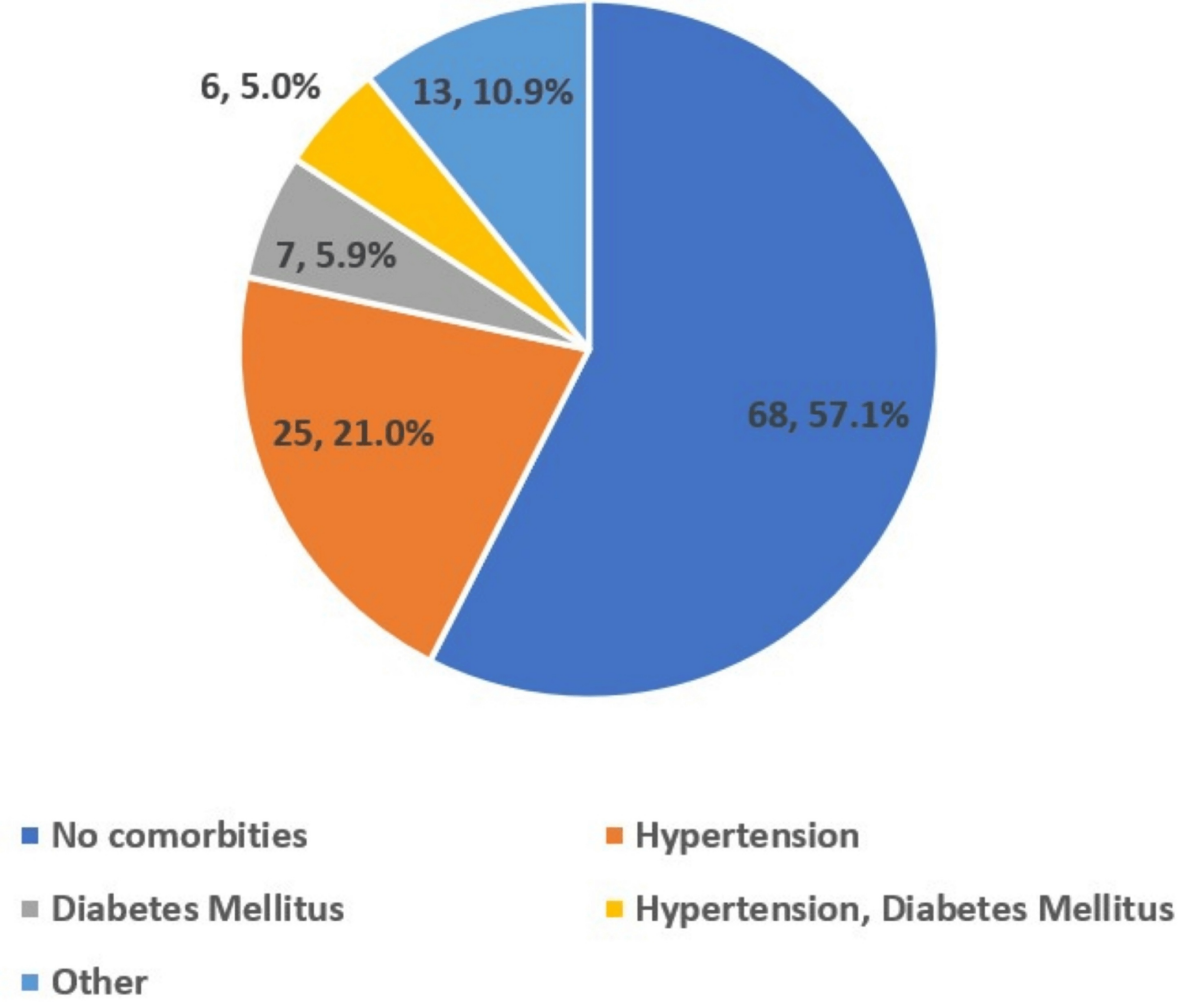
Distribution of common comorbidities among the study population (n=119)

Figure 1 illustrates the distribution of common comorbidities among the participants.

Osteoarthritis severity

**Figure 2:**
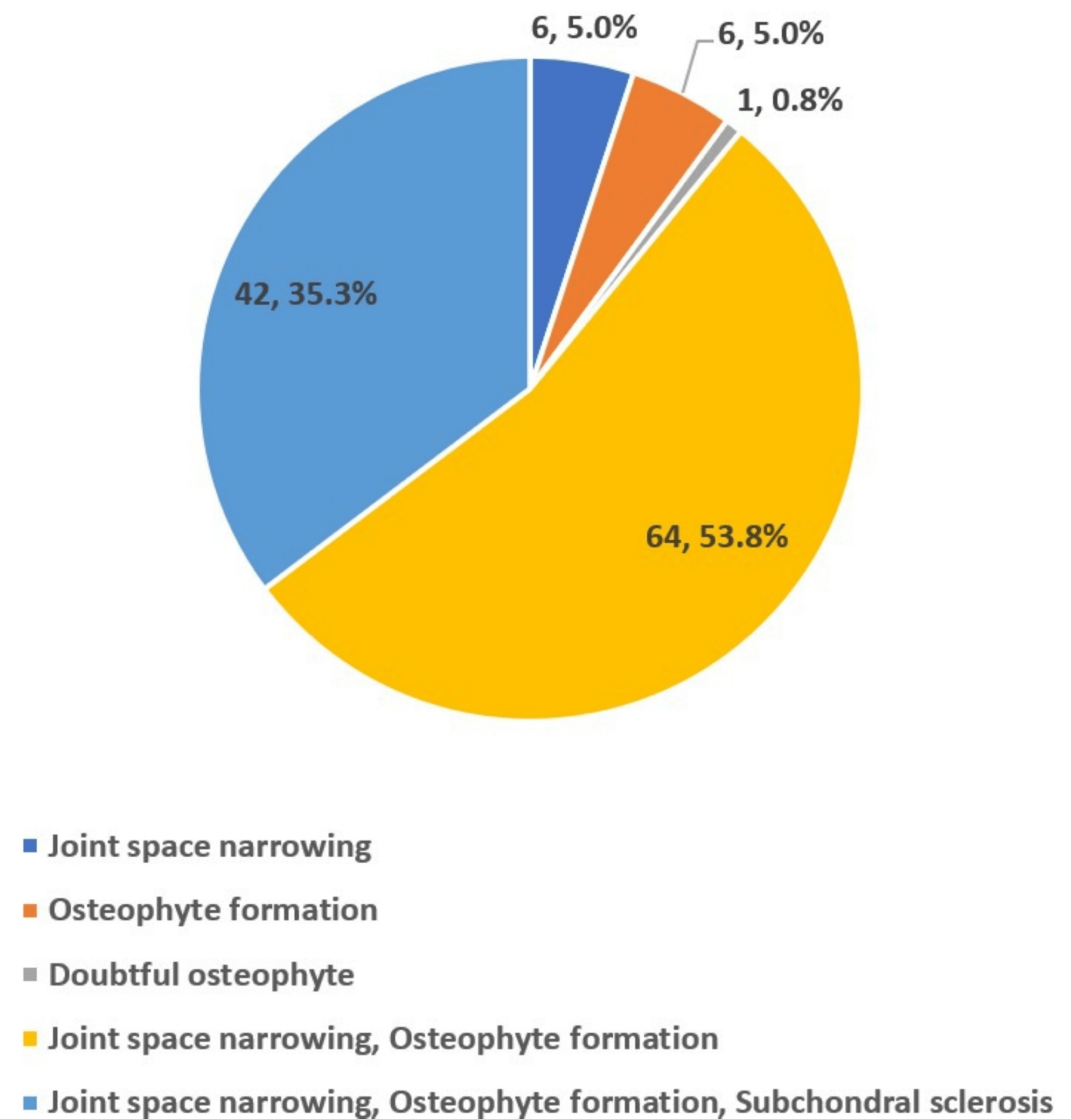
Graphical representation of findings of the sample population in knee X-ray (n=119)

Radiographic grading using the KL system showed that nine participants (7.6%) had grade 1, 50 (42.0%) had grade 2, 43 (36.1%) had grade 3, and 17 (14.3%) had grade 4 OA. Thus, 60 participants (50.4%) had moderate-to-severe OA (KL grades 3-4). Figure 2 displays the prevalence of radiographic features of OA observed on knee X-rays.

Fluoride exposure

Table 2 presents the fluoride concentration data. The mean serum fluoride level was 0.6±0.3 mg/L, the mean urinary fluoride was 1.0±0.6 mg/L, and the mean fluoride concentration in drinking water was 0.1±0.1 mg/L. A total of 97 participants (82.2%) consumed underground water, and only 21 of them (17.9%) reported using RO filtration for water sourced from rivers.

**Table 2:** Continuous variables showing various physical and clinical characteristics of the population (n=119) *KOOS used under the Creative Commons Attribution-NonCommercial-ShareAlike license from Roos EM, Lohmander LS. Health Qual Life Outcomes. 2003;1:64. VAS, visual analog scale; KOOS, knee injury and osteoarthritis outcome score

Variables	Mean	SD
Weight (kg)	63.3	11.6
Height (cm)	151.7	6.7
BMI (kg/m^2^)	27.4	4.6
Serum fluoride level mg/L	0.6	0.3
Urine fluoride level mg/L	1.0	0.6
Drinking water fluoride level mg/L	0.1	0.1
VAS score	3.6	1.7
KOOS score (n=118)*	48.8	15.6

Statistical associations

Water Source and OA Severity

Table 3 presents the chi-square analysis of categorical variables associated with KL grading. As shown in Table 3, participants consuming underground water had significantly higher KL grades (p=0.01).

**Table 3:** Chi-square test of variables with KL grading *P-values ≤0.05 were considered statistically significant. RO, reverse osmosis; OA, osteoarthritis; KL, Kellgren-Lawrence

Variables	Classification	KL grades 3 and 4	KL grades 1 and 2	Chi-square	P
Age	51-60	37	21	8.1	0.004*
	40-50	23	38		
Gender	Male	7	7	0.0	1.00
	Female	53	52		
Overweight/obese	Yes	51	47	0.6	0.45
	No	9	12		
Yadadri-Bhuvanagiri	Yes	33	29	0.5	0.47
	No	27	30		
Occupation	Unskilled	16	19	0.4	0.51
	Semi-skilled and above	44	40		
Drinking water	Underground	54	42	6.8	0.01*
	Others	6	17		
Water purification with RO	No	6	2	2.1	0.15
	Yes	54	57		
Diet	Mixed	59	57	0.4	0.55
	Veg	1	2		
Family H/o OA	Yes	19	11	2.5	0.11
	No	41	47		
Hypertensive/diabetes	Yes	23	22	0.0	0.91
	No	37	37		
Prolonged squatting/stair climbing	Yes	39	41	0.3	0.60
	No	21	18		

Age and OA Severity

Participants aged 51-60 years had increased odds of moderate-to-severe OA (OR: 2.9; 95% CI: 1.4-6.1). The significant variables from the chi-square analysis were taken for logistic regression. Table 4 summarizes the logistic regression findings.

**Table 4:** Regression of significant variables with KL grading KL, Kellgren-Lawrence; OR, odds ratios

Variables	Classification	KL grades 3 and 4	KL grades 1 and 2	OR	CI
Age (years)	51-60	37	21	2.9	1.4-6.1
	40-50	23	38	1	-
Drinking water	Underground	54	43	3.6	1.3-10.1
	Others	6	17	1	-

KL Grade and Fluoride Levels

Table 5 shows associations between fluoride biomarkers and categorical variables using the Mann-Whitney U test. Participants with KL grades 3-4 had significantly higher serum fluoride levels (p=0.007).

**Table 5:** Mann-Whitney U test of variables with various fluoride levels *P-values ≤0.05 were considered statistically significant. RO, reverse osmosis; OA, osteoarthritis; KL, Kellgren-Lawrence

Variables	Classification	P-values		
		Serum fluoride	Urine fluoride	Drinking water fluoride
Age	51-60	0.63	1.00	0.56
	40-50			
Gender	Male	0.35	0.50	0.15
	Female			
Overweight/obese	Yes	0.70	0.88	0.87
	No			
Yadadri-Bhuvanagiri	Yes	0.88	0.82	0.03*
	No			
Occupation	Unskilled	0.78	0.87	0.20
	Semi-skilled and above			
Drinking water	Underground	0.51	0.49	0.02*
	Others			
Water purification with RO	No	0.88	0.68	0.17
	Yes			
Diet	Mixed	0.11	0.08	0.69
	Veg			
Family H/o OA	Yes	0.63	0.56	0.94
	No			
Hypertensive/diabetes	Yes	0.73	0.84	0.27
	No			
Prolonged squatting/stair climbing	Yes	0.02*	0.04*	0.50
	No			
KL grading	3 and 4	0.007*	0.07	0.45
	1 and 2			

*Pain and Functional Scores*

Mann-Whitney U test results are shown in Table 6. Participants using RO-purified water reported significantly lower pain (VAS, p=0.02) and improved functional status (KOOS, p=0.05). KL grading was significantly associated with worse VAS pain scores (p<0.001) and KOOS functional scores (p<0.001).

**Table 6:** Mann-Whitney U test of variables with VAS and KOOS scores *P-values ≤0.05 were considered statistically significant. VAS, visual analog scale; KOOS, knee injury and osteoarthritis outcome score

Variables	Classification	P-values	
		VAS score	KOOS score
Age	51-60	0.16	0.23
	40-50		
Gender	Male	0.18	0.62
	Female		
Overweight/obese	Yes	0.64	0.07
	No		
Yadadri-Bhuvanagiri	Yes	0.81	0.26
	No		
Occupation	Unskilled	0.81	0.59
	Semi-skilled and above		
Drinking water	Underground	0.37	0.68
	Others		
Water purification with RO	No	0.02*	0.05*
	Yes		
Diet	Mixed	0.52	0.43
	Veg		
Family H/o OA	Yes	0.14	0.05
	No		
Hypertensive/diabetes	Yes	0.40	0.17
	No		
Prolonged squatting/stair climbing	Yes	0.44	0.92
	No		
KL grading	3 and 4	<0.001*	<0.001*
	1 and 2		

## Discussion

This study reveals a significant association between elevated fluoride exposure and increased radiographic severity of knee OA in a fluorosis-endemic population. Participants who consumed untreated underground water exhibited higher serum fluoride concentrations and more advanced KL grades. These findings reinforce previous reports from endemic regions of India that linked chronic fluoride exposure with degenerative joint pathology [12,17].

The biological plausibility of this relationship is supported by experimental and cellular studies demonstrating that excessive fluoride can induce chondrocyte apoptosis, disrupt extracellular matrix integrity, and promote oxidative stress, key mechanisms in OA progression [9,10,18]. Degenerative changes seen in OA models, such as cartilage thinning and matrix loss, parallel findings reported in animal studies involving fluoride exposure [19].

Our findings also underscore the additive effect of age and fluoride exposure. While OA is primarily age-related, chronic fluoride ingestion appears to amplify joint degeneration, suggesting a synergistic mechanism. Notably, patients aged 51-60 years were more likely to present with KL grades 3-4, and this association strengthened in the presence of elevated serum fluoride levels. This supports observations in earlier epidemiological studies where fluoride acted as a modifier of age-related joint deterioration [20].

The impact on clinical outcomes was evident: higher KL grades were significantly associated with worse pain (VAS) and functional scores (KOOS). These findings are consistent with other studies that identified musculoskeletal pain and stiffness as prominent features in populations exposed to high environmental fluoride, even without classical skeletal fluorosis [9,10]. A cross-sectional study by Singh et al. (2020) among knee arthritis patients also found significant differences in VAS score and KL grades between patients with elevated and normal fluoride levels [12]. A systematic review and meta-analysis by Wong et al. (2022) among adults aged 60 and above concluded that daily fluoride consumption, due to living in high-fluoride areas, was a risk factor for developing chronic lower back pain, with an adjusted OR of 1.58 (95% CI: 1.10-2.28) [21].

Importantly, water purification via RO was significantly associated with lower fluoride levels and improved clinical outcomes. Participants using RO-filtered water reported reduced pain and better functional scores, highlighting the public health value of household-level water treatment in fluoride-endemic areas. These findings align with earlier work suggesting RO filtration as a practical method for reducing fluoride intake and mitigating its systemic effects [20]. Community-based fluoride mitigation programs have demonstrated improved musculoskeletal health outcomes following installation of defluoridation units or provision of alternative safe water sources [22].

Although skeletal fluorosis is a well-defined clinical condition, its early or subclinical stages may mimic OA or coexist with it, leading to diagnostic uncertainty. Without fluoride biomarker evaluation, many such cases may be misclassified, resulting in inadequate management. Routine fluoride assessment should be considered in OA workups in endemic regions, particularly when patients present with early-onset or atypical joint degeneration. This approach may help refine differential diagnosis and guide appropriate community-level interventions.

Limitations

This study has several limitations. First, its cross-sectional design precludes the establishment of causality between fluoride exposure and OA progression. Second, although we measured fluoride biomarkers in serum, urine, and water, we did not assess chronicity or longitudinal fluctuations in fluoride levels. Third, relevant confounding factors such as dietary calcium intake, bone mineral density, and levels of physical activity were not evaluated, which may influence OA severity and fluoride metabolism. Then, the absence of a comparative control group from a non-endemic region limits our ability to generalize these findings beyond similar geographic and environmental contexts. Finally, there was a potential for selection bias due to consecutive hospital OPD recruitment.

## Conclusions

This study highlights a significant association between elevated fluoride biomarker levels and greater radiographic and symptomatic severity of knee OA in a fluorosis-endemic region. Individuals exposed to untreated underground water exhibited higher fluoride levels and worse clinical outcomes. These findings support the inclusion of environmental fluoride monitoring in public health and clinical strategies for OA management. Promoting access to safe drinking water, especially through RO purification, may mitigate fluoride-related joint damage and reduce the OA burden. Future longitudinal studies are essential to validate these findings and explore potential interventions.

## References

[1] Hunter DJ, Bierma-Zeinstra S (2019). Osteoarthritis. Lancet.

[2] Bijlsma JWJ, Berenbaum F, Lafeber FP (2011). Osteoarthritis: an update with relevance for clinical practice. Lancet.

[3] Safiri S, Kolahi AA, Smith E (2020). Global, regional and national burden of osteoarthritis 1990-2017: a systematic analysis of the Global Burden of Disease Study 2017. Ann Rheum Dis.

[4] Mobasheri A, Batt M (2016). An update on the pathophysiology of osteoarthritis. Ann Phys Rehabil Med.

[5] Choubisa SL (2001). Endemic fluorosis in southern Rajasthan, India. Fluoride.

[6] (2025). WHO. Guidelines for drinking-water quality, 4th edition, incorporating the 1st addendum. https://www.who.int/publications/i/item/9789241549950.

[7] Reddy DR (2009). Neurology of endemic skeletal fluorosis. Neurol India.

[8] (2025). Susheela AK. A treatise on fluorosis. New Delhi: Fluorosis Research and Rural Development Foundation. New Delhi: Fluorosis Research and Rural Development Foundation.

[9] Johnston NR, Strobel SA (2020). Principles of fluoride toxicity and the cellular response: a review. Arch Toxicol.

[10] Chauhan D, Kumar R (2025). Molecular Mechanism of Fluoride-Induced Toxicity and Associated Health Hazards. Fluorides in Drinking Water.

[11] Dou H, Wang S, Hu J, Song J, Zhang C, Wang J, Xiao L (2023). Osteoarthritis models: from animals to tissue engineering. J Tissue Eng.

[12] Singh VK, Rathore KS, Khan G, Rahim A, Rashid A, Chauhan S (2020). Clinical and radiological study of serum fluoride in relation to knee osteoarthritis. Malays Orthop J.

[13] Kellgren JH, Lawrence J (1957). Radiological assessment of osteo-arthrosis. Ann Rheum Dis.

[14] Huskisson EC (1974). Measurement of pain. Lancet.

[15] Roos EM, Lohmander LS (2003). The knee injury and osteoarthritis outcome score (KOOS): from joint injury to osteoarthritis. Health Qual Life Outcomes.

[16] Frant MS, Ross JW Jr (1966). Electrode for sensing fluoride ion activity in solution. Science.

[17] Shukla A (2016). Skeletal fluorosis mimicking seronegative arthritis. Indian J Rheumatol.

[18] Wang M, Luo K, Sha T (2024). Apoptosis and inflammation involved with fluoride-induced bone injuries. Nutrients.

[19] Zhou X, Zu W, Zhao L, Gong X, Jiang Q (2025). Sodium fluoride accelerates apoptosis, oxidative stress and matrix degradation of condylar chondrocytes by upregulating MMP-13 and RANKL. Am J Transl Res.

[20] Sowanou A, Meng X, Zhong N (2022). Association between osteoarthritis and water fluoride among Tongyu residents, China, 2019: a case-control of population-based study. Biol Trace Elem Res.

[21] Wong CK, Mak RY, Kwok TS (2022). Prevalence, incidence, and factors associated with non-specific chronic low back pain in community-dwelling older adults aged 60 years and older: a systematic review and meta-analysis. J Pain.

[22] Susheela AK, Toteja GS (2018). Prevention & control of fluorosis & linked disorders: Developments in the 21st Century - Reaching out to patients in the community & hospital settings for recovery. Indian J Med Res.

